# Roles of Gut Microbial Metabolites in Diabetic Kidney Disease

**DOI:** 10.3389/fendo.2021.636175

**Published:** 2021-05-20

**Authors:** Qing Fang, Na Liu, Binjie Zheng, Fei Guo, Xiangchang Zeng, Xinyi Huang, Dongsheng Ouyang

**Affiliations:** ^1^ Department of Clinical Pharmacology, Xiangya Hospital, Central South University, Changsha, China; ^2^ Institute of Clinical Pharmacology, Central South University, Hunan Key Laboratory of Pharmacogenetics, Changsha, China; ^3^ Engineering Research Center of Applied Technology of Pharmacogenomics, Ministry of Education, Changsha, China; ^4^ National Clinical Research Center for Geriatric Disorders, Changsha, China; ^5^ Hunan Key Laboratory for Bioanalysis of Complex Matrix Samples, Changsha Duxact Biotech Co., Ltd., Changsha, China

**Keywords:** gut microbiota, microbial metabolites, diabetic kidney disease, diabetes, short-chain fatty acids, Trimethylamine-N-oxide

## Abstract

Diabetes is a highly prevalent metabolic disease that has emerged as a global challenge due to its increasing prevalence and lack of sustainable treatment. Diabetic kidney disease (DKD), which is one of the most frequent and severe microvascular complications of diabetes, is difficult to treat with contemporary glucose-lowering medications. The gut microbiota plays an important role in human health and disease, and its metabolites have both beneficial and harmful effects on vital physiological processes. In this review, we summarize the current findings regarding the role of gut microbial metabolites in the development and progression of DKD, which will help us better understand the possible mechanisms of DKD and explore potential therapeutic approaches for DKD.

## Introduction

With the continuous improvement of people’s living standards, changes in lifestyle, and environmental factors, the incidence of diabetes mellitus (DM), which is a metabolic disorder, has been increasing year by year. According to data released by the International Diabetes Federation (IDF), about 463 million people worldwide were living with DM in 2019. The global prevalence of DM is expected to rise to rise to 10.2% (578 million people) by 2030 and to 10.9% (700 million people) by 2045 (1). Patients with DM are more prone to serious complications that contribute to increased mortality and reduced quality of life. About 30-40% of patients with DM develop diabetic kidney disease (DKD), which is one of the major complications of DM, and most cases progress to end-stage renal disease (2).

The hyperglycemic condition initiates multiple events that damage the kidney structurally and functionally, such as glomerular hyperfiltration, proteinuria, thickening of the glomerular basement membrane, mesangial matrix accumulation, podocyte damage and glomerulosclerosis (3). Renal hemodynamics changes, the renin-angiotensin-aldosterone system, oxidative stress, inflammatory responses and fibrosis are also major factors in the pathogenesis of DKD (4). Current therapy for DKD includes antihypertensive and antiproteinuric means, as well as the use of angiotensin receptor blockers and angiotensin converting enzyme inhibitors. In addition, sodium glucose cotransporter 2 (SGLT-2) inhibitors and glucagon-like peptide 1 (GLP-1) receptor agonists are novel diabetes medications that prevent kidney failure (5). However, these means have limited efficacy in preventing the progression of DKD (6). The complicated pathogenesis of DKD has not yet been elucidated. Understanding the pathophysiology of DKD is crucial for its prevention and treatment.

Recent advances in high-throughput metagenomic sequencing technologies have increased our knowledge of the symbiotic relationship between the gut microbiota and its host (7). The gut microbiota, as an important environmental factor, has emerged as a crucial regulator of human health and disease (8–10). The metabolites produced by the gut microbiota may also have pathogenic or beneficial effects on the host. These metabolites and their end products may play key roles in the host’s metabolic network (11), immune processes (12) and neurobiological processes (13).

Recently, metabolomic studies have found that the microbial metabolite profile is altered in patients with type 2 diabetes (T2D) (14) and DKD (15). Mounting evidence supports the critical role of the gut microbiota as a factor (either beneficial or harmful) in the development of T2D (11) and DKD (16). Moreover, a number of metabolites are produced by the gut microbiota, using dietary nutrients as precursors, suggesting that diet has an important impact on gut microbial metabolites. In this review, we offer a summary of short-chain fatty acids (SCFAs), trimethylamine-N-oxide (TMAO), bile acids (BAs), polyphenols, tryptophan-derived metabolites, branched-chain amino acids (BCAAs) and other metabolites that play important roles in the pathogenesis and progression of DKD. We also discuss the potential mechanisms of microbial metabolites and DKD.

### Short-Chain Fatty Acids

SCFAs, including acetate, propionate, and butyrate, are produced by the microbial community through the fermentation of non-digestible carbohydrates. Acetate and propionate are mainly produced by Bacteroidetes, whereas butyrate is primarily generated by Firmicutes (17). SCFAs can be used by the host for the biosynthesis of lipids, cholesterol, and proteins or as an energy source by gut mucosal cells (18). The effects of SCFAs are in part mediated by G-protein coupled receptors (GPR41, GPR43, and GPR109A) and histone deacetylase (HDAC), which are related to oxidative stress, immune, and inflammatory responses (19–22).

SCFAs have been reported to have multiple beneficial regulatory roles in both type 1 diabetes (T1D) (23, 24) and T2D (25). Several studies have also indicated their important roles in DKD (**Table 1**). SCFAs, especially acetate and butyrate, inhibit oxidative stress and inflammation in mouse glomerular mesangial cells that have been induced by high glucose and lipopolysaccharides (26). Treatment with a high-fiber diet or directly treatment with SCFAs can both protect against the development of DKD in mice by regulating the key pathways and genes involved in innate immunity, inflammation, and macrophage recruitment. GPR43 and GPR109A are critical to SCFAs-mediated protection against DKD (31). Exogenous sodium butyrate administration can improve DKD by reducing inflammation and oxidative stress, and by ameliorating fibrosis, apoptosis, and DNA damage, and this has been proven in different animal models (19, 22, 31). Exogenous sodium butyrate can also could protect human glomerular mesangial cells against high glucose-induced pyroptosis (32). These studies suggest that SCFAs, especially butyrate, may act as potential therapeutic targets for DKD.

**Table 1:** Beneficial and Harmful effect of SCFAs on DKD *in vivo* and *vitro*.

Supplement	Animal/Cell Type	Mechanism	References
SCFAs (acetate, butyrate, propionate)	Mouse glomerular mesangial cells (SV40-MES-13)	(+) GPR 43; oxidative stress (ROS↓, MDA↓, SOD↑) ↓; inflammation (ICAM-1↓, MCP-1↓, IL-1β↓) ↓	(26)
High-fiber diet, SCFAs (acetate, butyrate, propionate)	C57BL/6, Gpr43-/- and Gpr109A-/- mice; Mouse kidney tubular epithelial cells and podocytes;	(+) GPR43 and GPR109A; IL-6↓, IFNγ↓, CCL2↓, CXCL10↓; fibronectin↓, TGFβ↓	(23)
SCFAs (acetate, butyrate, propionate)	C57BL/6 mice; Mouse glomerular mesangial cells (SV40-MES-13)	(+) GPR43-β-arrestin-2 signaling; oxidative stress (ROS↓) ↓; NF-κB inflammatory signaling↓	(27)
NaB	SD rats	(-) HDACs; eNOS↓, iNOS↓; α-SMA↓, collagen I↓, fibronectin↓, TGF-β_1_↓; NF-κB↓; apoptosis↓; DNA damage↓	(22)
NaB	C57BL/6 and Nrf2-/- mice	(-) HDACs; (+) NRF2	(28)
NaB	Human renal glomerular endothelial cells	(-) caspase 1-GSDMD canonical pyroptosis pathway; (-) NF-κB/IκB-α signaling pathway	(29)
NaB	db/db and db/m mice; Mouse mesangial cells (SV40-MES-13)	(-) micro7a-5p/P311/TGF-β_1_ pathway	(30)
NaB	Normal rat kidney tubular epithelial (NRK−52E) cells	(-) HDAC2; oxidative stress (ROS↓, SOD↑, LDH↓) ↓	(20)

(+), active; (-), inhibit; SCFA, short−chain fatty acids; NaB, sodium butyrate; GPR 43, G-protein-coupled receptor 43; HDAC, histone deacetylase; NRF2, nuclear factor erythroid 2-related factor 2; GSDMD, gasdermin D; ROS, reactive oxygen species; MDA, Malondialdehyde; SOD, superoxide dismutase; ICAM-1, intercellular cell adhesion molecule-1; MCP-1, monocyte chemotactic protein-1; IL-1β, interleukin-1 β; IL-6, interleukin-6; TGFβ, transforming growth factor-β; eNOS, endothelial nitric oxide synthase; iNOS, inducible nitric oxide synthase; α-SMA, α-smooth muscle actin; LDH, lactate dehydrogenase.

Although much evidence suggests that increased butyrate production benefits the host through antidiabetic effects, some studies have suggested that acetate may exacerbate DKD. In some animal models, plasma acetate levels have been reported to be positively correlated with the intrarenal angiotensin II protein, which has long been considered to be one of the initiators of DKD. Acetate might also be involved in the kidney injury of early DKD (33). In addition, acetate has been reported to dysregulate cholesterol homeostasis through activating GPR43, thereby contributing to the tubulointerstitial injury of DKD (34). Whether SCFAs production is beneficial or harmful when it comes to DKD remains to be further studied.

### Trimethylamine-N-Oxide

Dietary choline, phosphatidylcholine, and L-carnitine are metabolized into trimethylamine (TMA) by intestinal commensal bacteria. A choline-utilization gene cluster (Cut) responsible for anaerobic conversion of choline to TMA was identified in the sulfate-reducing bacterium *Desulfovibrio desulfuricans*. *CutC and CutD*, which are crucial genes in the cluster, encoding for choline TMA-lyase and its activating protein. Moreover, in some bacteria of the gender *Acinetobacter and Serratia*, *CntA and CntB* gens encode the two subunits of the oxidoreductase enzyme necessary to convert L-carnitine into TMA (35). On the other hand, a *YeaW/YeaX* gene pair encodes some oxygenase and oxidoreductase enzymes with substrate promiscuity for betaine, γ-butyrobetaine, choline and L-carnitine. These genes, not only *CntA/CntB* and *YeaW/YeaX*, but also orthologs and homologs of them, can be found in a wide range of gut microbiota: Actinobacteria, Betaproteobacteria (*Achromobacter*), Firmicutes (*Sporosarcina*), and Gammaproteobacteria (*Citrobacter, E.coli, Klebsiella pneumoniae, Providencia, and Shigella*) (36).

Once TMA is produced, most TMA is oxidized into TMAO in the liver predominantly by the enzyme flavin-containing monooxygenase 3 (37). Some clinical studies have investigated the role of TMAO in predicting the prognostic outcomes and mortality of diverse diseases (38–40). In addition to its predictive value, TMAO has been implicated in the pathogenesis of various human diseases, including cardiovascular (41), kidney (42), metabolic (43) and neurological (44) disorders.

TMAO is linked to the pathogenesis of many diseases by activating inflammatory pathways, such as nucleotide-binding domain, leucine-rich-containing family, pyrin domain-containing-3 (NLRP3) inflammasome signals (45–48) and nuclear factor-κB signals (48–50), resulting in the release of inflammatory cytokines. Moreover, some studies have indicated that TMAO and its precursor can contribute to the pathogenesis of cardiovascular diseases by inducing endothelial dysfunction (51–54). In addition, both inflammation and endothelial dysfunction play important roles in the pathogenesis of DKD.

**Figure 1:**
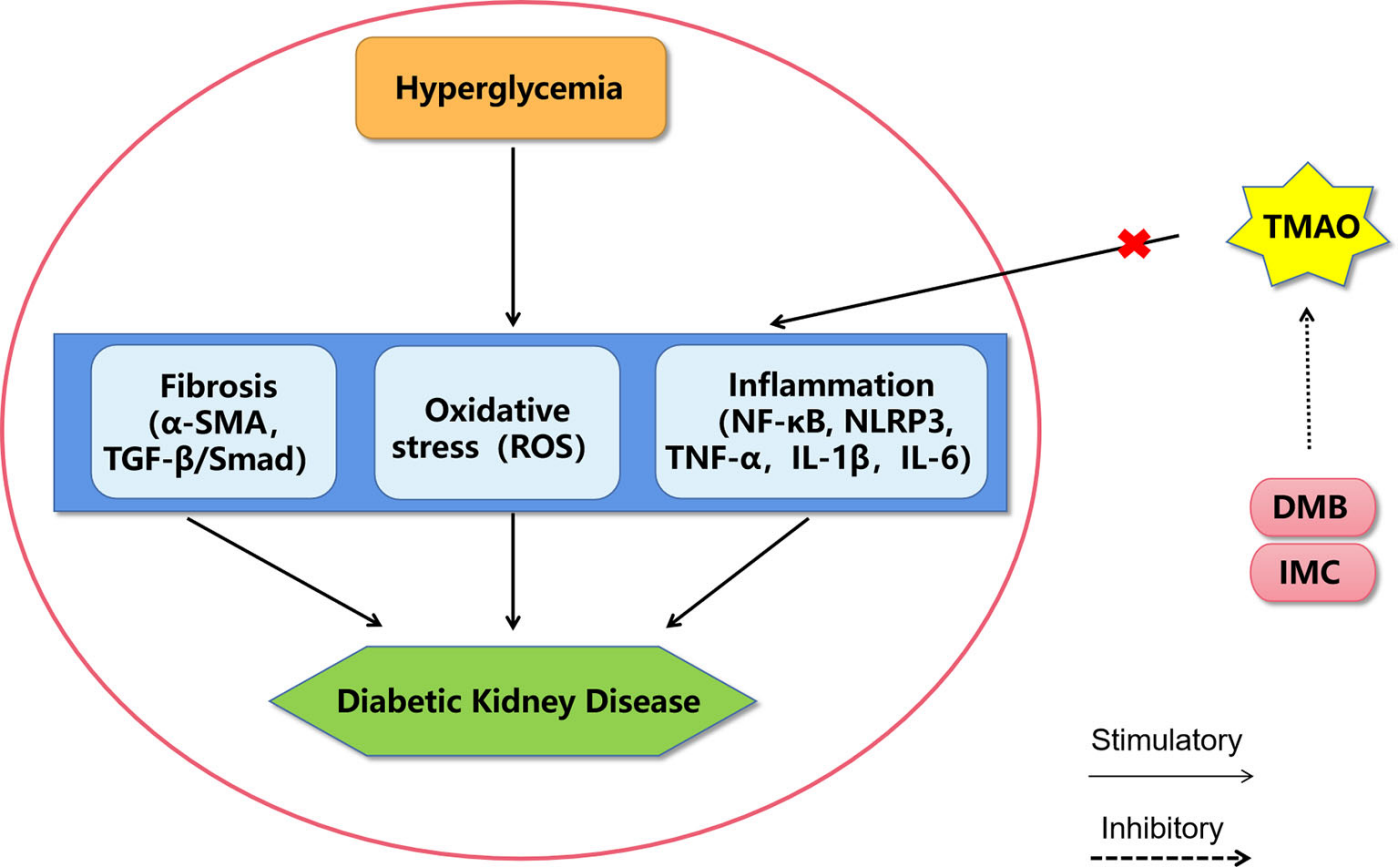
A proposed model of diabetic kidney disease mediated by TMAO. The mechanism of TMAO promoting the progression of diabetic kidney disease may be through promoting inflammation, oxidative stress and fibrosis in renal system. The choline TMA lyase inhibitor DMB and IMC may improve diabetic kidney disease by inhibiting TMAO levels. DMB, 3,3-dimethyl-1-butanol; IMC, iodomethylcholine; NF-κB, nuclear factor kappa-light-chain-enhancer of activated B cells; NLRP3, Nod-like receptor pyrin domain 3 inflammasome, TNF-α, tumor necrosis factor α; IL-1β, interleukin-1β; IL-6, interleukin-6; ROS, reactive oxygen species; α-SMA, alpha sarcomeric actin; TGF-β, transforming growth factor-β.

Recently, it has been widely accepted that increased levels of circulating TMAO directly contribute to renal dysfunction by promoting inflammation, oxidative stress, and fibrosis. Both TMAO-supplemented and choline-supplemented mice have shown elevated TMAO levels, which were associated with increases in tubulointerstitial fibrosis and collagen deposition (42). In a mouse model of high-fat-diet-induced obesity, elevated TMAO levels were found to promote renal oxidative stress and inflammation, subsequently contributing to renal interstitial fibrosis and dysfunction (55). In rats with chronic kidney disease (CKD), elevated TMAO levels promote vascular oxidative stress and inflammation, contributing to endothelial dysfunction (56). In a CKD mouse model, dietary supplementation with either choline or TMAO was found to significantly augment multiple indices of renal functional impairment and fibrosis (57). Supplementation with 3,3-dimethyl-1-butanol (an inhibitor of trimethylamine formation) or iodomethylcholine (an inhibitor of prototypic mechanism-based gut microbial choline TMA-lyase) can reduce plasma TMAO levels and prevent adverse renal structural and functional alterations in animal models (55–57). Therefore, high TMAO levels may exacerbate DKD, and TMAO inhibitors may have therapeutic potential to ameliorate DKD (**Figure 1**).

The regulation of TMAO in the DKD still warrants more investigation. Despite growing interest in TMAO biology, the receptor for TMAO is not yet known. Recent evidence has shown that TMAO directly bounds to and activates the protein kinase R-like endoplasmic reticulum kinase, which is an endoplasmic reticulum stress kinase (43). The next milestone is to identify the direct targets of TMAO.

### Bile Acids

BAs are host-microbial co-metabolites and important signaling molecules. The primary BAs produced by the host can be metabolized by the gut microbiota into a secondary BAs (58). When a small portion of unabsorbed BAs enter the distal ileum, cecum, and colon, they undergo various reactions *via* microbiota: deconjugation, dehydroxylation, oxidation and epimerization reactions (59, 60). BA deconjugation is driven by bile salt hydrolase, which have been identified in *Bacteroides*, *Bifidobacterium*, *Clostridium*, *Enterococcus*, and *Lactobacillus* (61). Dehydroxylation occurs after deconjugation, and is catalyzed by members of the Firmicutes phylum, including Clostridium (*C. scindens* or *C. hylemonae*) and *Eubacterium*. Oxidation and epimerization require BA hydroxysteroid dehydrogenases produced by intestinal *Bacteroides*, Firmicutes (including *Clostridium*, *Eubacterium*, and *Ruminococcus*), and *Escherichia* (60).

BAs are endocrine signaling molecules that affect host physiology *via* the activation of BA receptors. The two major BA receptors that regulate the host metabolism are the nuclear farnesoid X receptor (FXR) and the membrane-bound Takeda G protein-coupled receptor 5 (TGR5) (62). Both FXR and TGR5 have protective roles in DKD (63).

Renal expression of FXR is predominantly tubular and less prominently glomerular, mesangial, and podocytal (64). FXR expression is decreased in people with diabetes- and obesity-related kidney disease. In a series of rodent models of diabetes, the expression levels of FXR and its target genes were found to be downregulated in the kidney (65). Supplementation with FXR agonists, such as tauroursodeoxycholic acid, has been shown to attenuate glomerular and tubular injury in db/db mice and diabetic endothelial nitric oxide synthase-deficient mice (66). Moreover, the FXR agonist GW4064 can improve the functional and structural changes in the kidney of db/db mice (67).

TGR5 expression and activity is impaired in the kidneys of humans and rodents with obesity and diabetes (68). TGR5 activation reduces the renal inflammatory reactions in diabetic mice, thereby improving renal fibrosis (69). In high glucose-treated glomerular mesangial cells, TGR5 activation was found to significantly decrease the expression levels of transforming growth factor beta 1 and fibronectin, which can both accelerate renal fibrosis (70, 71).

**Figure 2:**
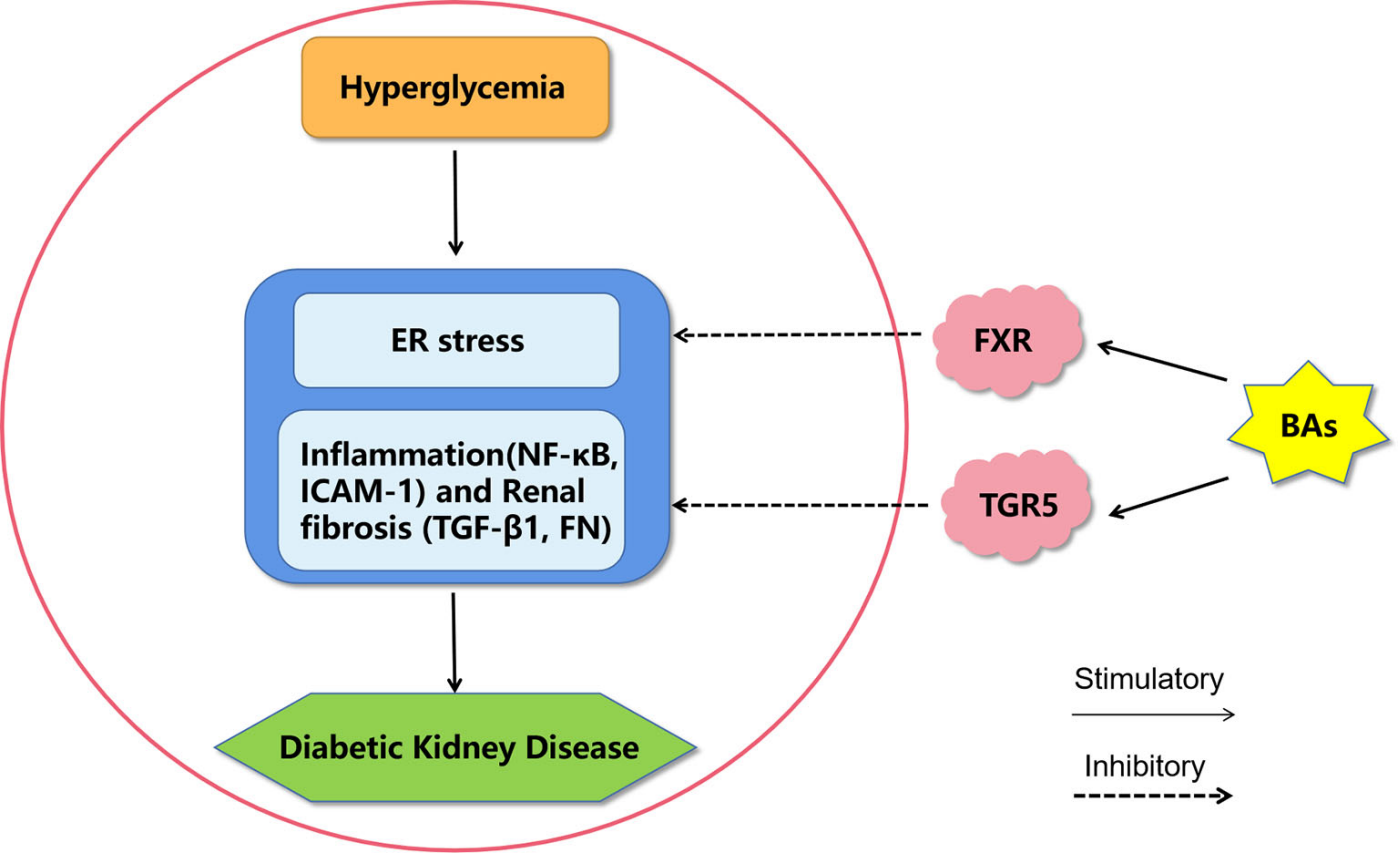
A proposed model of diabetic kidney disease mediated by bile acids. Bile acids may inhibit endoplasmic reticulum stress, inflammation and fibrosis by activating FXR and TGR5 to improve diabetic kidney disease. BAs, bile acids; FXR, Farnesoid X receptor; TGR5, G protein-coupled receptor 5; ER stress, endoplasmic reticulum stress; NF-κB, nuclear factor kappa-light-chain-enhancer of activated B cells; ICAM-1, intercellular adhesion molecule-1; TGF-β1, transforming growth factor-β1; FN, fibronectin.

The BA signaling pathway plays an extremely important role in T2D and DKD, and it is an important target for drug intervention (**Figure 2**). BAs have been used directly to treat diabetes and obesity. Metformin, which is a first-line antidiabetic drug, acts in part through the intestinal FXR axis to improve T2D. Oral synthetic FXR antagonists may be of potential translational value in the clinical treatment of T2D (72) Moreover, both BA sequestrants and apical sodium-dependent BA transporter inhibitors can reduce BA absorption and have a therapeutic effect on T2D by activating FXR (73). In the future, semisynthetic BA analogues for the treatment of T2D and DKD need more focus.

### Protein-Bound Uremic Toxins

Protein-bound uremic toxins, such as indoxyl sulfate (IS), p-cresyl sulfate (pCS), p-cresyl glucuronide (pCG), and phenyl sulfate, originate from the gut microbial metabolism of the aromatic amino acids, tyrosine, phenylalanine, and tryptophan. These uremic toxins have been associated with cardiovascular disease and mortality in CKD, and several uremic toxins have also been found to exert toxic effects in the kidney. The levels of these uremic toxins are elevated in T2D patients who progress to end-stage kidney disease (74, 75) and elevated levels of these uremic toxins increase the risk of progression to end-stage kidney disease in patients with T2D (74).

IS is derived from tryptophan metabolism. Tryptophan is digested by intestinal bacteria (*E. coli*, *Proteus vulgaris*, *Paracolobactrum coliforme*, *Achromobacter liquefaciens*, and *Bacteroides* spp) to indole, and it is metabolized to IS in the liver (76). Increasing levels of IS are correlated with changes in albuminuria and the estimated glomerular filtration rate, and they are associated with the progression of DKD in patients with T1D and T2D (77–79), as well as in animal models of DM (80–82). IS also can directly induce tubulointerstitial injury, renal oxidative stress and inflammation in mice that undergone nephrectomy (83, 84), as well as in human renal proximal tubular epithelial (HK-2) cells (85, 86).

Both pCS and pCG originate from the intestinal microbial fermentation of tyrosine into p-cresol, and p-cresol is subsequently conjugated to either sulfate or glucuronic acid resulting in the formation of pCS or pCG, respectively (87). The intestinal bacteria generating p-cresol mainly belong to the families Bacteroidaceae, Bifidobacteriaceae, Clostridiaceae, Enterococcaceae, Eubacteriaceae, Fusobacteriaceae, Lachnospiraceae, Lactobacillaceae, Porphyromonadaceae, Staphylococcaceae, Ruminococcaceae, and Veillonellaceae (88). The levels of pCS and pCG are elevated in patients with CKD, and pCG can cause phenotypical changes in renal proximal tubule cells (89). In addition, pCS can directly influence cell viability and induce cell death (90, 91). It has also been reported that pCS can induce reactive oxygen species and inflammatory cytokines in 5/6 nephrectomized rats and HK-2 cells (92).

Phenyl sulfate is produced by the metabolism of the amino acid tyrosine by the gut microbiota. First, phenol is synthesized in the gut and then metabolized into phenyl sulfate by the liver. The intestinal bacteria generating phenol mainly belong to the families Clostridiaceae, Enterococcaceae, Staphylococcaceae, Bacteroidaceae, Bifidobacteriaceae, and Enterobacteriaceae (93, 94). Phenyl sulfate is then secreted by proximal tubular cells through the action of SLCO4C1, which is the only organic acid transporter polypeptide in the human kidney (95). In a cohort of diabetic patients, phenyl sulfate levels were found to be statistically significantly correlated with both basal albuminuria and the 2-year progression of DKD. Further, phenyl sulfate was found to directly induce albuminuria *via* podocyte damage in diabetic animal models (15).

**Figure 3:**
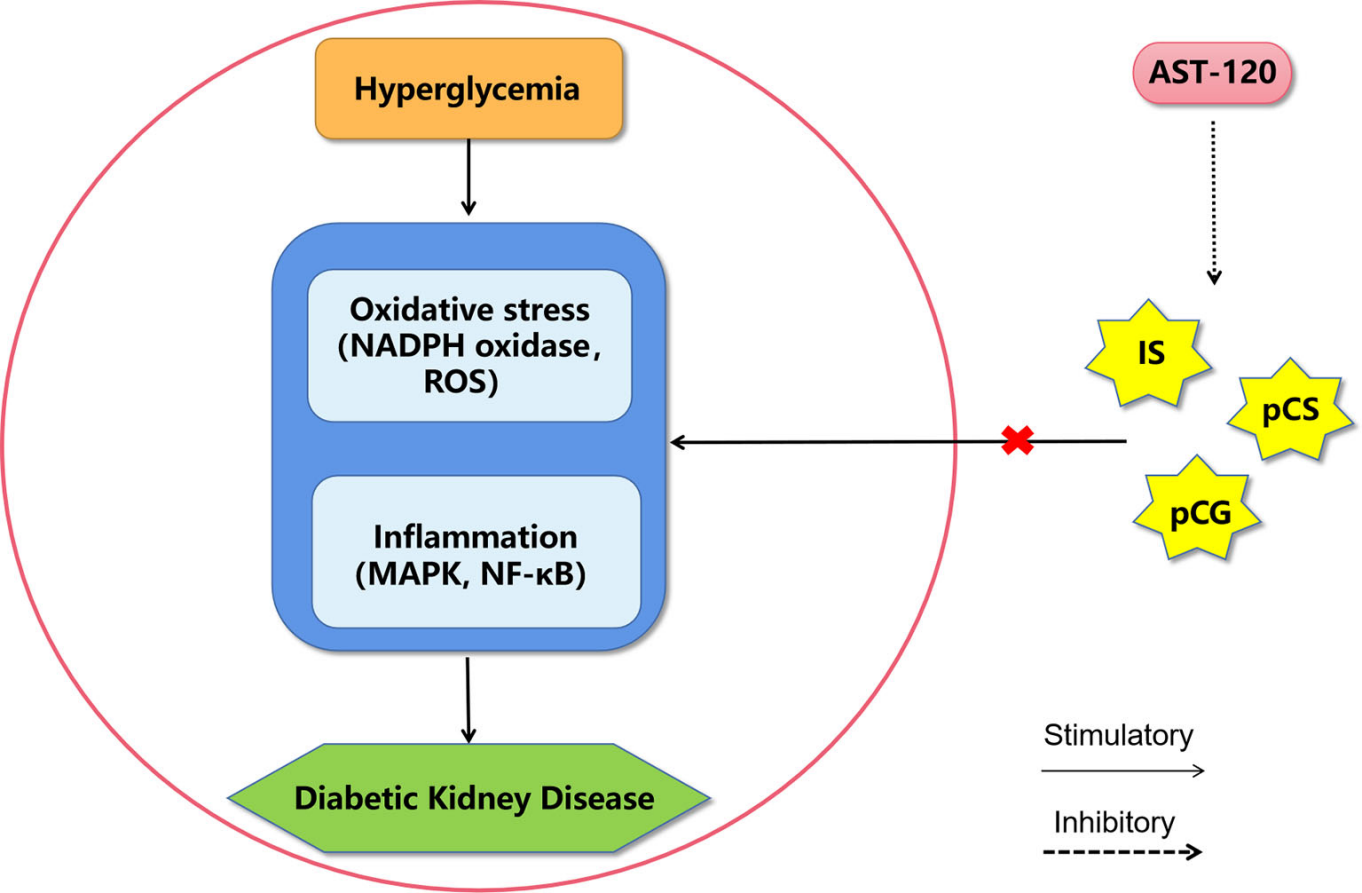
A proposed model of diabetic kidney disease mediated by protein-bound uremic toxins. The mechanism of protein-bound uremic toxins promoting the progression of diabetic kidney disease may be through promoting inflammation and oxidative stress in renal system. AST-120 may improve diabetic kidney disease by removing serum and urinary uremic toxins. IS, Indoxyl sulfate; pCG, p-cresyl glucuronide; pCS, p-cresyl sulfate; ROS, reactive oxygen species; MAPK, mitogen-activated protein kinase; NF-κB, nuclear factor kappa-light-chain-enhancer of activated B cells.

These protein-bound uremic toxins are not only markers of the risk for DKD occurrence in diabetic patients, but they are also risk factors that directly facilitate the development of DKD (**Figure 3**). However, the molecular mechanisms of these uremic toxins in DKD still need further study. Some drugs that target uremic toxins, such as AST-120 (Kremezin), which is an oral adsorbent, protect against the progression of both DKD (84, 96) and CKD (97, 98) by removing serum and urinary uremic toxins.

### Polyphenols-Derived Microbial Metabolites

Polyphenols are produced in plants and have excellent antibacterial, antifungal, antioxidant, and photo-protective properties (99). Natural polyphenols, such as ellagitannins, lignans, isoflavones, and flavanones, which are poorly absorbed or not absorbed at all. Interaction with gut microbiota leads to the biochemical transformations of the native phytochemicals into more bioavailable metabolites. These gut microbiota transformations are grouped into three major catabolic processes: hydrolysis (O-deglycosylations and ester hydrolysis), cleavage (C-ring cleavage; delactonization, demethylation), and reductions (dehydroxylation and double bond reduction) (100). *Lactobacillus*, *Bifidobacterium*, *Bacteroides*, *Enterococcus*, *Enterobacter*, and Firmicutes have been demonstrated to participate in hydrolysis (101–103). Cleavage reactions are catalyzed by *Clostridium*, *Coriobacteriaceae*, *Eubacterium* and *Eggerthella* strains (101, 104). *Gordonibacter urolithinfaciens* and *Lactonifactor longoviformis* catalyze different reduction reactions (105–107).

Enterolactone and enterodiol, which are therapeutically relevant polyphenols, are formed as the secondary gut bacterial metabolites of lignans. Urinary levels of enterolactone are associated with lower risk of developing T2D in women in the United States (108). Pre-diagnostic enterolactone concentrations are inversely associated with all-cause and diabetes-specific mortality (109). Enterolactone was found to increase glucose uptake in an AMPK-dependent manner in L6 myotubes and to improve glucose tolerance in db/db mice (110). Many studies have reported that enterolactone may have some benefit for T2D. However, there are also some opposite conclusions. One study did not find a significant association between urine enterolactone levels and T2D risk in Chinese adults (111). Moreover, enterolactone was found to enhanced the hepatic insulin resistance *via* increased sphingolipid concentrations in the palmitate-rich condition of HepG2 cells (112). Thus, the relationship between enterolactone and T2D should be further studied.

Urolithin A (UA), which is a main gut microbiota-derived metabolite of pomegranate ellagitannins, plays a direct role in improving systemic insulin sensitivity (113). In addition, UA can prevent high-fat-diet-induced insulin resistance and glucose intolerance in mice (114). Further, UA can effectively improve β-cell dysfunction, possibly by regulating autophagy and the AKT/mTOR signaling pathway in the pancreas of diabetic mice (115). Moreover, UA has shown a protective effect in some acute kidney injury animal models *via* modulation of inflammation, oxidative stress, autophagy, and apoptosis (52, 116–118), which are also some of the main mechanisms of DKD. Thus, UA may be capable of attenuating DKD. Urolithin C (UC), which is another microbiota ellagitannin metabolite, is a glucose-dependent activator of insulin secretion *via* the facilitation of the opening of L-type Ca^2+^ channels in β-cells (119). UC can also influence β-cell function by affecting the activation of intracellular signaling proteins, specifically ERK1/2 (120). Urolithin also has been reported have some certain benefits for diabetes complications. Urolithins (includes urolithin A, B, C, D and urolithin B-3-O-glucuronide) may exert positive effects in modulating the pro-inflammatory mediators and growth factors by rat cardiac myocytes and fibroblasts exposed to high glucose concentrations (121). In streptozotocin-induced diabetic rat, both urolithin A and B administration may be able to prevent the initial inflammatory response of myocardial tissue to hyperglycemia and the negative impact of the altered diabetic milieu on cardiac performance (122).

Taken together, polyphenols might have differential microbial metabolites that may improve T2D and its complications. Thus, it is crucial to understand the bacterial pathways involved in the metabolism of polyphenols and their specific roles in T2D and DKD.

### Branched-Chain Amino Acids

BCAAs, including leucine, isoleucine, and valine, are among the nine essential amino acids synthesized by the gut microbiota. The food sources most enriched in BCAAs are meat, fish, dairy products, and eggs (123).

BCAAs are known as biomarkers for insulin resistance and predictors of diabetes development (124). In a recent prospective cohort study, BCAAs showed a strong association with early risk of T2D (125). The serum metabolomes of insulin-resistant and T2D individuals are characterized by increased BCAAs levels (126). Higher intake of total dietary BCAAs, leucine and valine in particular, may increase the incidence of insulin resistance by more than 60% in adults and play an important role in the development of diabetes (127). In contrast, short-term reduction of dietary BCAAs may acutely decrease meal-induced insulin secretion, and improve postprandial insulin sensitivity (128). Furthermore, in both animal and cell models, excess BCAAs have been found to result in liver insulin resistance (129, 130). These results suggest that BCAAs may not only be biomarkers, but also causal agents of insulin resistance and T2D. Evidence of a causal role of BCAAs in human diabetes has also been suggested in some genetic studies. In a mendelian randomization analysis, researchers used genome-wide association studies coupled with large-scale metabolomic measurements to investigate the aetiologic relationship between BCAA metabolism and T2D. They suggested that BCAA-raising polymorphisms were associated with a higher risk of T2D (131). In addition, genetic evidence suggests that genetically elevated insulin resistance is associated with higher concentrations of all BCAAs, supporting the idea that BCAA metabolism lies on a causal pathway from adiposity and insulin resistance to T2D (132). Another study showed that higher BCAA levels do not have a causal effect on insulin resistance while increased insulin resistance drives higher fasting levels of circulating BCAAs (133).

In T2D patients with stages 1 or 2 CKD, high serum BCAA levels are independently associated with a decline in the estimated glomerular filtration rate (134). BCAAs can also directly influence renal function. In one animal model, 5/6 nephrectomized rats receiving a BCAA diet showed a decrease in the estimated glomerular filtration rate and an increase in smooth muscle actin and collagen mRNA expression levels, suggesting renal dysfunction, greater inflammation, and fibrosis in the kidney (135).

However, several studies have suggested that BCAAs may be an effective means of preventing and treating DM and DKD. Moderate intake of BCAA-rich protein was found to improve glucose homeostasis in mice fed a high-fat diet (136). In streptozotocin-induced diabetic rats, treatment with a low dose of BCAAs was found to recover islet function (137). Moreover, one study found that BCAAs countered oxidative stress in the kidney of diabetic rats and alleviated diabetic kidney injury (138), while another study showed that BCAAs protected renal mesangial cells from high-glucose-induced stress (139). More studies are needed to clarify the relationships between BCAAs and T2D. Furthermore, the factors resulting in the differences among these studies needed to be identified.

Many bacterial species are capable of regulating biosynthesis, transport, and metabolism of BCAAs (140). Moreover, transplantation of the microbiota from obese humans to germ-free mice causes a significant increase in circulating BCAAs (141). Additional studies are needed to quantify the microbiota-derived BCAAs in the circulating pool of these metabolites.

### Other Metabolites

Imidazole propionate, which is a microbially produced amino acid-derived metabolite, is present at higher concentrations in people with T2D. A study demonstrated that imidazole propionate directly impaired glucose tolerance and insulin signaling through mTORC1, meaning that imidazole propionate may contribute to the pathogenesis of T2D (142).

4-Cresol is a product of the colonic fermentation of tyrosine and phenylalanine, and it has been reported to be related to T2D. Serum concentrations of 4-cresol are inversely correlated with T2D. The chronic administration of nontoxic doses of 4-cresol was found to reduce adiposity and glucose intolerance in an animal model, and 4-cresol was found to stimulate insulin secretion and β-cell proliferation *in vivo* and *in vitro* (143, 144). 4-Cresol may be a regulator of T2D endophenotypes and have potential therapeutic applications.

The metabolism of dietary and host-derived sulfur-containing compounds to hydrogen sulfide (H_2_S) by the gut microbiota has many prominent connections to host health and disease (145). H_2_S potentially has both beneficial and toxic effects (146). The plasma H_2_S levels of Japanese patients with T2D were found to be reduced and significantly associated with their hemoglobin A1c levels (147). H_2_S also has been suggested to be linked with GLP-1, which has become an important therapeutic target in the treatment of T2D and obesity. By supplementing with a prebiotic chondroitin sulfate, mice were found to enhanced GLP-1 and insulin secretion, improved oral glucose tolerance, and reduced food consumption, suggesting that H_2_S plays a stimulatory role in GLP-1 secretion (148). However, chronic administration of H_2_S-releasing agents was found to increase serum glucose, decrease glucose tolerance, and decrease insulin secretion in rats with T2D (149). In the future, studies are needed to clarify the effects of different doses of H_2_S in T2D. As for DKD, H_2_S has been reported to have a protective effect and may be used as a novel therapeutic agent.

Hippuric acid is a gut microbial mammalian co-metabolite of benzoic acid; it is subsequently conjugated with glycine in the mitochondria, and then excreted in the urine (150). Several metabolomics studies have shown that hippuric acid levels in patients with impaired glucose tolerance and diabetes are lower than those in healthy people (151). Hippuric acid has also been proposed as a potential urinary biomarker for fruit, vegetable, and polyphenol consumption. Consumption of bilberries and flavonoids, which can increase hippuric acid levels, is associated with a favorable risk factor profile for T2D and better glucose and insulin metabolism (152, 153). Further, hippuric acid has been reported to be decreased in both human diabetic renal pathology studies (154) and animal DKD models (155), and it has been suggested as an additional indicators of DKD. However, there is no evidence to excluded the direct effect of hippuric acid on T2D or DKD. Further studies are needed to infer causality between hippuric acid and T2D or DKD.

## Therapeutic Drugs for DKD and Gut Microbial Metabolites

In general, none of the widely used current treatments specifically address the underlying molecular processes responsible for DKD. Several interventional strategies have involved multifactorial approaches, including blood pressure and glucose lowering (156).

RAAS inhibitors have been the treatment of choice for DKD, following the publication of clinical trial results demonstrating benefits of angiotensin converting enzyme inhibitors and angiotensin receptor blockers for decreasing albuminuria in patients with DKD (157). Metformin, the most frequently administered medication to decrease blood glucose, has recently been suggested to enrich SCFA-producing microbiota, such as such as *Blautia*, *Bacteroides*, *Butyricoccus*, *Bifidobacterium*, *Prevotella*, *Megasphaera*, *Butyrivibrio* (158). SGLT2 inhibitors, GLP-1 receptor agonists, and dipeptidyl peptidase 4 (DPP4) inhibitors are three new classes of glucose­lowering agents for patients with DKD (159–161). SGLT2 inhibition has been reported to promote elevations in the levels of the SCFAs acetate and butyric acid in cecal contents of hypertensive mice (162). Vildagliptin, a DPP4 inhibitor, has been reported to enrich SCFA-producing bacteria (163). Moreover, mice on HFD with DPP-4 inhibitor PKF-275-055 treatment showed enriched butyrate-producing *Rumminococcus* and of the acetogen *Dorea* (164).

Some drugs for the treatment of DKD can increase the level of SCFA by enriching SCFA-producing microbiota. Whether the mechanism of these drugs in the treatment of DKD is mediated by gut microbial metabolites remains unknown. The relationship between these drugs and gut microbial metabolites still needs more research.

## Conclusions and Future Perspectives

**Figure 4:**
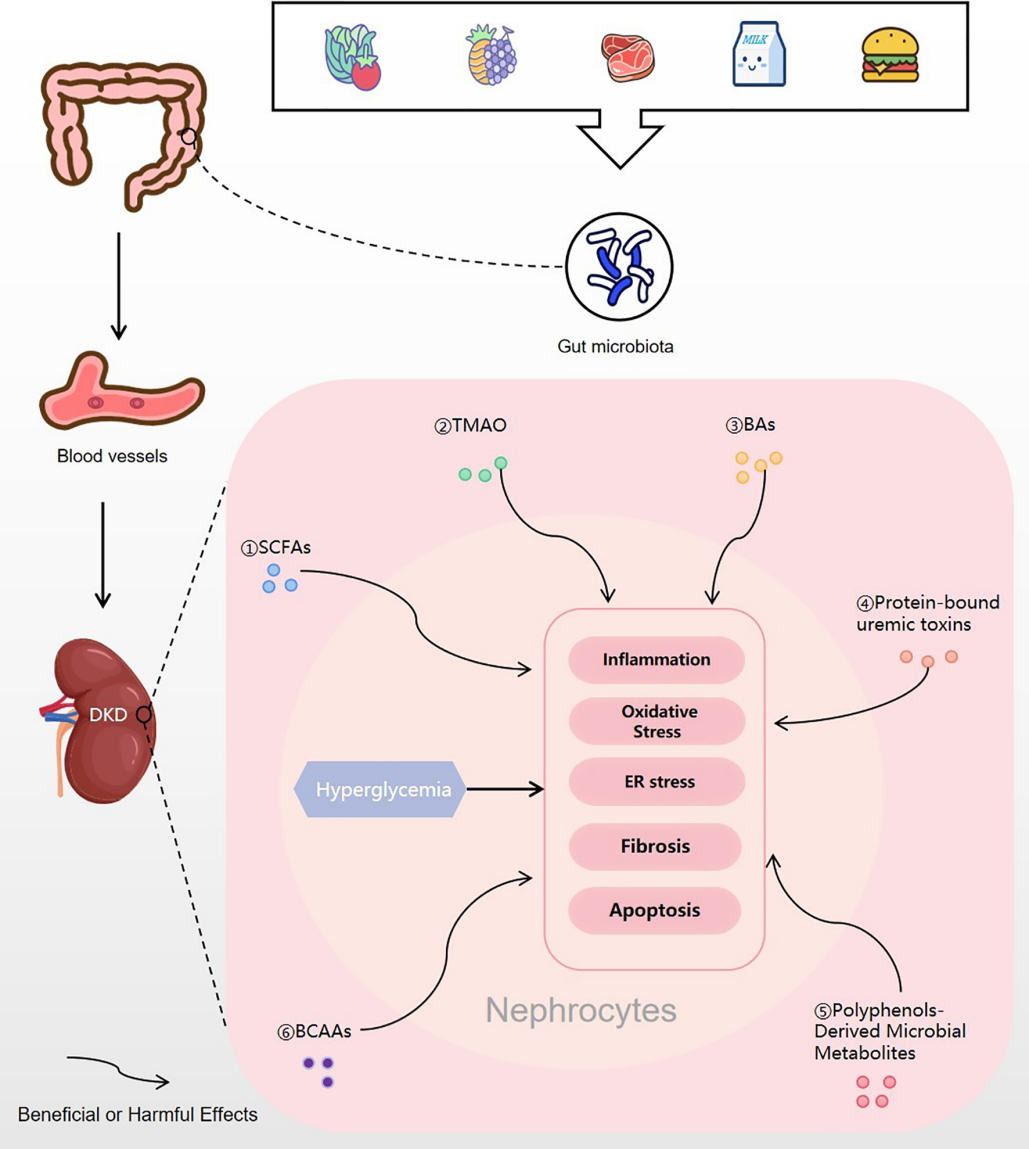
Gut microbial metabolites regulating DKD. Food is digested and absorbed by the gastrointestinal tract, and various gut microbial metabolites are produced under the action of the gut microbiota. Then, the microbial metabolites are absorbed into the blood vessels and finally enter the kidney. In nephrocytes, gut microbial metabolites of SCFAs, TMAO, BAs, protein-bound uremic toxins, polyphenols-derived microbial metabolites and BCAAs may trigger or inhibit inflammation, oxidative stress, ER stress, fibrosis or apoptosis, which will improve or excacerbate the progress of DKD. DKD, diabetic kidney disease; SCFA, short-chain fatty acid; TMAO, trimethylamine N-oxide; BA, bile acids; BCAA, branched-chain amino acid; ER stress, endoplasmic reticulum stress.

There is growing evidence of the roles of gut microbial metabolites as biomarkers of the pathophysiological features or as pathogenic agents of DKD. In this review, we highlighted aspects relating to the involvement of microbiota metabolites in the pathogenesis of DKD (**Figure 4**). In the future, we should continue to look for microbial metabolites that can be used to diagnose and treat DKD. However, there are still many challenges to be overcome on the path toward using microbial metabolites for therapies.

First, most microbial metabolites act as signaling molecules by binding to receptors and triggering downstream signaling cascades. Therefore, we should identify the targets of these microbial metabolites in humans. Some metabolites, such as SCFAs and BAs, interact with G-protein-coupled receptors (GPCRs) associated with diverse functions. Many studies have shown that large-scale functional screening for GPCRs can identify microbial metabolites that exert various physiological functions by activating GPCRs. There are many undiscovered receptors similar to GPCRs, and efforts should be made to identify these receptors and generate drugs targeting these receptors in host tissues.

Second, most studies to date have only clarified the correlations between microbial metabolites and T2D, and lack of research on causality. Large prospective cohort studies are needed to determine if microbial metabolites are altered prior to or after disease onset. The result of some studies showing causal effects in rodents should be confirmed in humans. If the results on causality can be confirmed in humans, then further research on the human intestinal microbiota may lead to the development of novel diagnostic and therapeutic tools.

Finally, the concentrations of intestinal metabolites might be highly context-dependent. If the metabolite is beneficial to human health, it needs to be supplemented once its level falls below the physiological level. The supplementation strategy needs to consider the route and frequency of administration, individual differences in pharmacokinetics, and side effects for doses that exceed physiological concentrations. If the metabolite contributes to the pathophysiology of the disease, then the production of the metabolite needs to be suppressed. Inhibiting related enzymes that produce metabolites is a promising method. Developing inhibitors targeting bacterial enzymes is another therapeutic strategy to prevent the action of harmful microbial metabolites, as in the case of TMA lyase producing TMA, a precursor for TMAO. Metabolites do not act in isolation, and thus the combined signals mediated by different metabolites need to be investigated.

We believe that numerous challenges must be overcome on the path toward using microbial metabolites for therapies. Despite these difficulties, the metabolic pathways involved in the production and signaling of microbiome-related metabolites are huge untapped opportunities to regulate disease susceptibility.

## Author Contributions

QF, NL, and BZ performed the literature search. QF drafted the manuscript. XZ, FG, and XH provided critical intellectual contributions. DO directed the research and made the critical revision. All authors contributed to the article and approved the submitted version.

## Funding

This work was supported by the National Development of Key Novel Drugs for Special Projects of China [grant numbers 2017ZX09304014], the Hunan Key Laboratory for Bioanalysis of Complex Matrix Samples [grant number 2017TP1037], the Hunan Province Science and Technology Innovation Project, Significance of TMAO, a metabolite of intestinal flora, in adverse cardiovascular reactions caused by antibiotics [grant number 2018SK52008], the Key R&D Programs of Hunan Province [grant number 2019SK2241], and the International Scientific and Technological Innovation Cooperation Base for Bioanalysis of Complex Matrix Samples in Hunan Province [grant number 2019CB1014].

## Conflict of Interest

Author DO was employed by company Changsha Duxact Biotech Co., Ltd.

The remaining authors declare that the research was conducted in the absence of any commercial or financial relationships that could be construed as a potential conflict of interest.
